# Messaging to Reduce Booster Hesitancy among the Fully Vaccinated

**DOI:** 10.3390/vaccines12091066

**Published:** 2024-09-18

**Authors:** Chao Qin, Susan Joslyn, Jee Hoon Han, Sonia Savelli, Nidhi Agrawal

**Affiliations:** 1 Psychology Department, University of Washington, Seattle, WA 98195, USA; robertqc@uw.edu (C.Q.); jhan326@uw.edu (J.H.H.); 2 Human-Centered Design & Engineering, University of Washington, Seattle, WA 98195, USA; ssavelli@uw.edu; 3 Foster School of Business, University of Washington, Seattle, WA 98195, USA; nidhia@uw.edu

**Keywords:** science communication, booster hesitancy, trust in science, decision making, risk

## Abstract

Vaccine hesitancy was a serious problem in the United States throughout the COVID-19 pandemic, due in part to the reduction in public trust in science that accompanied the pandemic. Now we are facing a new, similar but more extensive problem: booster hesitancy. Even fewer Americans are current on the mRNA booster. We present the results of an experiment with residents of the US who received all initial doses of the mRNA vaccine but who were not up to date on the booster. Participants read a scientific explanation describing either the safety or the effectiveness of the boosters or nothing in the control group. The explanations significantly increased (compared to the control) participants’ perceptions of the safety and effectiveness of the mRNA booster, as well as their willingness to get boosted. Explanations also improved emotions and attitudes toward the booster. Furthermore, although liberals were significantly more willing to get boosted than were conservatives, improvement due to the explanations was similar across political ideology groups. However, when interactions were observed, conservatives increased to a greater degree. Importantly, the explanations increased participants’ perception of scientists’ expertise and knowledge, as well as participants’ trust in scientists and the vaccine technology.

## 1. Introduction

Vaccine hesitancy was a serious problem in the United States throughout the COVID-19 pandemic. Almost a third of Americans failed to get fully vaccinated for COVID-19, and most of those remain unvaccinated to this day [1]. Several reasons have been suggested for this situation, including those associated with demographics [2,3,4] and political ideology [5,6,7], as well as distrust in the science [8], distrust in the mRNA testing process [9,10], and an incomplete understanding of the safety and efficacy of the novel mRNA vaccines among the vaccine hesitant [11].

However, what is potentially even more puzzling is that, even though booster shots are clearly necessary as vaccine efficacy wanes over time and the virus evolves away from the current vaccine target [12,13], many of those who are fully vaccinated are hesitant to receive the booster shots. This is despite the fact that Americans have long been familiar with, although not completely accepting of, the need for boosters for evolving viruses such as those made annually available for the flu [14]. Indeed, there is some evidence that those who are hesitant to get the mRNA vaccine booster tend to be those who have not received a flu shot in the previous year [15]. However, while about 50% of Americans were up to date on their flu vaccinations in the 2022–2023 season, only about 17% of Americans were up to date on the COVID-19 booster shot [16]. In other words, 75% of those who got the complete series of mRNA vaccine initial doses and most of those who got a recent flu shot had not received the most recent mRNA vaccine booster for which they are eligible.

To date, the research devoted to the question of COVID-19 booster hesitancy has been inconclusive. This is partially because the majority was conducted prior to widespread booster availability and, in some cases, among those who had not had the complete set of initial doses, as well as those who had. Thus, unsurprisingly, this research suggests that booster hesitancy in the US is related to general hesitancy about the mRNA vaccines [17]. Indeed, many vaccinated with the initial doses may have done so reluctantly and due to social and/or workplace requirements that were in place at the time rather than genuine willingness. Therefore, these groups may be overlapping to some extent. Moreover, there is some evidence that the need for boosters may deepen hesitancy for the initial doses by decreasing the perceived efficacy of the mRNA vaccine overall [18].

In addition, there is evidence that booster hesitancy in the US is related to side effects experienced after the initial doses [19], as well as other perceived risks of the booster [17]. Indeed, perceived risk of the COVID-19 vaccine has been a major issue from the outset (e.g., [9,11,20,21]). Safety issues that were cited include not only the potential for dangerous side effects and allergic reactions [22] but also concerns regarding the testing and rapid development of the mRNA vaccine, as well as lack of trust in the testing process (e.g., [9,10,11]). Booster hesitancy could be due to similar concerns, as well as concerns about the cumulative effects of additional doses of the vaccine. Clearly, vaccine safety has been a primary concern throughout the process.

There is also some evidence that a contributor to booster hesitancy is the perception among some that boosters are not necessary [19]. This may be a trust issue, as the initial booster recommendations came from the pharmaceutical companies and may have been perceived as economically motivated. Some may believe that the initial vaccine doses provide sufficient protection. In addition, booster hesitancy could be due to the notion that the COVID-19 threat has passed, despite that fact that, as of this writing, almost twice as many Americans die each year from COVID-19 as from the common flu [1]. Whatever the reason, booster hesitancy may stem from the fact that some people perceive less risk from the disease and therefore regard booster doses as unnecessary.

In sum, although there is an abundance of evidence that the mRNA vaccines are effective and that there is a clear need for boosters for which safety is well documented, we are now faced with a new problem, booster hesitancy. The goal of the research presented here was to gain a better understanding of what contributes to and mitigates COVID-19 booster hesitancy. Here, we test interventions comprising scientific explanations that address two prominent issues likely contributing to current hesitancy, concerns about vaccine safety, and incomplete understanding of vaccine effectiveness and the need for boosters.

Understanding vaccine safety and effectiveness may be crucial stumbling blocks because the technology and testing process that generated the COVID-19 mRNA vaccines and boosters, as well as the statistics that describe their safety and effectiveness, are complex and may be challenging for non-experts to understand. For that reason, explanations using everyday language that include the relevant details and address known misunderstandings may help people to realize that the boosters are safe, necessary to sustain immunity, and effective in doing so. Two such explanations were tested in the experiment reported below (see Table 1). Notice that, although the explanations are written in non-technical language, they also include relevant statistics that speak to known concerns. Recent research suggests that members of the public have a “working understanding” of numerical information such as this and can use it to make better decisions [23,24,25,26]. Interestingly, the benefits of numerical information were observed regardless of level of education [27]. Similarly, a better understanding of the scientific issues related to the safety and effectiveness of the COVID-19 vaccines and boosters, including the relevant statistics, may increase willingness to get the booster. In fact, there is some evidence that scientific explanations such as these were able to reduce hesitancy for the initial doses [11].

However, it may not be as simple as providing an explanation, because booster hesitancy, like vaccine hesitancy in general, may not be due to insufficient understanding alone. Attributing attitudes like vaccine hesitancy entirely to insufficient understanding is known as the “deficit model” and criticized by many for discounting the role of social, cultural, and psychological factors that have been shown to play a role in risk perceptions and attitudes [28,29]. Indeed, the issue of vaccination, as well as other protective measures against COVID-19, became highly politicized [21,22,30,31,32], with conservatives being generally less willing to engage in protective measures than liberals, despite, in some cases, having comparable risk perception [11,33]. Therefore, it may be that booster hesitancy, like vaccine hesitancy, is an identity-relevant issue for some [34,35]. Therefore, ideology was measured in the experiment reported here to determine whether it interacted with the manipulations. In other words, explanations may be effective among liberals but not among conservatives. In fact, they may backfire among conservatives, known as a “boomerang effect” [35].

Similarly, and often linked to ideology, is the issue of trust in science, which may be necessary to accept new vaccines and their boosters. There is now evidence that explanations that provide scientific information, including the relevant statistics, even those that address highly charged issues, such as mRNA vaccines and climate change, increase trust in the information provided [11,36]. This is presumably the case because the explanations were perceived as honest and forthcoming rather than merely persuasive. Therefore, we also sought to gauge the impact of the explanations tested in the experiment reported here on trust and emotions related to the boosters, as well as understanding and willingness to get the booster.

Other psychological factors may play a role as well. For instance, vaccine hesitancy might have to do with whether the choice one makes is active or passive. Some vaccine-hesitant individuals may anticipate greater regret for a negative outcome that results from an active choice on their part (getting vaccinated and experiencing side effects) than from refraining to act (remaining unvaccinated and contracting COVID-19). This effect, known as omission bias [37], was also examined to determine whether it interacted with the explanations.

In sum, the experiment described below tested two simple but complete explanations, written in everyday language, describing the statistics that speak to both the safety and effectiveness of the COVID-19 booster shots to determine whether they increased understanding and reduced vaccine hesitancy. We also tested factors that might be related to willingness to get the booster or might be affected by the explanations, such as risk perceptions, emotions, trust in science, susceptibility to omission bias, and political ideology.

## 2. Materials and Methods

### 2.1. Participants

A total of 1409 participants who were fully vaccinated with the initial doses of the mRNA vaccine but who reported not receiving all eligible boosters were recruited in October 2022 from Prolific Academic, a crowdsourcing platform for online research. Each participant was paid USD 2 for participation. The mean age was 37 years (SD = 12.85, range from 18 to 89 years). There were 642 (46%) females, 738 (52%) males, and 29 (2%) others. The dataset was fairly representative of the US population, with an over-representation of Asian Americans and liberals, in addition to a slightly higher average education level. See Appendix A for additional demographic information.

### 2.2. Procedure

Participants were recruited on 13 October 2022, when the bivalent booster, which provided protection against the Omicron variant of COVID-19, was available in the US [38]. Eligible participants (residents of the US and vaccinated with at least one dose) were redirected to the experiment, hosted on Qualtrics. Informed consent was obtained from all participants involved in the study. Prior to reading the explanations, all participants answered seven questions (see Appendix B) to gauge their baseline risk perception and emotions about COVID-19. Participants responded to the first six of these questions (Q1–6) by moving a marker on a Visual Analog Scale (VAS), with anchors described in Appendix B.

Participants then indicated their vaccine status by answering the following question (Q7, Appendix B): “Choose the statement that best describes your status with regard to the mRNA COVID-19 booster shots”. There were four options: (1) I am not fully vaccinated with the initial doses of the vaccine, and, therefore, I am not eligible for booster shots. (2) I am fully vaccinated with the initial doses, but I have received none of the mRNA COVID-19 booster shots for which I am eligible. (3) I am fully vaccinated with the initial doses, and I have already received some but not all mRNA COVID-19 booster shots for which I am eligible. (4) I am fully vaccinated with the initial doses, and I have already received all the mRNA COVID-19 booster shots for which I am eligible. Only the 1409 participants who chose option (2) or (3) were included in the analysis reported below.

Then, participants were randomly assigned to one of three conditions (see Table 1), two of which provided experimental explanations. One condition (effectiveness) explained that, while the initial doses of the mRNA vaccine were effective in the first five months, their effectiveness dropped in the sixth month [12,38,39]. Therefore, boosters were needed for continued effective protection. Whether this recommendation was coming from the CDC or from the pharmaceutical companies was manipulated between groups. However, this manipulation did not lead to significant differences in any of the major dependent variables, so these conditions were combined in the analyses below. The second experimental condition, the safety condition, explained that a majority of the side effects of the booster were minor and were the result of the vaccine stimulating the immune system [40]. There was also a no-information control condition in which no explanation was given. Please note that all the information provided in the explanations was accurate and based on the most recent scientific evidence at the time.

Then, participants who indicated that they were fully vaccinated but not fully boosted were shown explanations based on their experimental condition, or none in the control condition. All participants then answered 18 post-explanation questions (Q8–25 in Appendix B. The first question (Q8; adapted from [9,11,29]) asked was as follows: “If you have not received all the mRNA COVID-19 booster shots for which you are eligible, choose the statement that best describes your situation or intentions”. There were four options: (1) I would get an (or another if I already have one) mRNA COVID-19 booster shot as soon as possible. (2) I would wait to get an (or another if I already have one) mRNA COVID-19 booster shot until there is more information. (3) I would not get an (or another if I already have one) mRNA COVID-19 booster shot. (4) I don’t know.

Participants responded to the next 15 questions by moving a marker on a VAS [11,41]. These were designed to gauge the direct impact of the explanations on participants’ perceptions of the safety and effectiveness of the booster was well other on factors that might contribute to hesitancy such as, trust and emotions (see Appendix B). First, participants rated the perceived safety (Q9) and effectiveness of booster in protecting them from COVID-19 (Q10–12). The effectiveness questions included effectiveness in protecting them from contracting COVID-19, directly addressed in the explanation, as well as related consequences, such as becoming severely ill or dying from COVID-19 and contracting new variants of COVID-19. Responses to these questions were expected to be affected by explanations. Participants in the experimental conditions also rated their trust in the explanations (Q13), trust in the scientists who developed the booster (Q14), and perceptions of the expertise and understanding of the scientists who developed the booster (Q15–16). To determine whether any lack of trust was due to the perception that the process had been rushed, participants also rated the degree to which they agreed with the statement that the vaccine-testing procedure had been compromised (Q17). Concerns about safety and effectiveness, short-term side effects, and long-term side effects were also rated (Q18–20). Participants then rated their emotions related to the booster, including fear, anxiety, and hope (Q21–23). In addition, participants rated their trust in the mRNA vaccine and traditional vaccines technologies (Q24 and Q25).

Finally, a question was asked to gauge whether omission bias influenced participants’ booster intentions (Q26). Participants indicated which of the following situations would cause more regret: (1) you chose to get the booster and suffered severe side effects; (2) you chose not to get the booster and suffered from severe illness due to COVID-19; (3) same amount of regret in both cases; and (4) no regret.

In order to determine whether participants understood the slightly more complex efficacy explanation, they were asked additional questions (Appendix B), including the likelihood of getting COVID-19 in the first three months (Q27) and after six months (Q28) following the initial doses of the mRNA vaccine. They were also asked to rate the effectiveness of the vaccine in the first five months (Q29). Finally, they rated the likelihood of getting COVID-19 after receiving the booster (Q30). In all cases, those with the effectiveness explanation rated lower likelihood of infection and greater effectiveness with the vaccine and booster, respectively, than in the control or safety conditions. The unregistered analyses for these manipulation-check questions are reported in Appendix C.

Next, participants provided demographic information (e.g., gender, age, and political ideology; see Q31–36, Appendix B). These data are reported in Appendix A. Then, a multiple-choice attention-check question (Q37) instructed participants to choose Ebola instead of COVID-19, with the options (1) COVID-19, (2) cancer, (3) Ebola, and (4) diabetes.

### 2.3. Design

The experiment used a single-factor between-groups design. The independent variable *explanation* had three levels: no-information control, effectiveness explanation, and safety explanation (see Table 1). A Chi-square test of independence indicated that the proportion of political ideology groups was not significantly different by explanation condition (χ^2^(4) = 2.09, *p* = 0.72).

## 3. Results

The primary goal of the research presented here was to examine the impact of the explanations on intentions to get fully boosted (Q8) and on participants’ perception of the issues targeted in the explanations, booster safety (Q9), and effectiveness (Q10–12). We also examined whether the explanations impacted other potential contributors to booster hesitancy, including trust in the scientists who developed the booster (Q14), the perceived expertise and understanding of the scientists (Q15–16), trust in the technology of mRNA and conventional vaccines (Q24, Q25), the belief that the testing procedure had been compromised (Q17), concerns about the booster (Q18–20), other emotions related to the booster (Q21, Q22, and Q23), and source of regret (Q26).

The hypotheses were that both explanations (compared to the no-information control) would do the following:

1. Increase intentions to get the booster;
2. Increase targeted perceptions (safety/effectiveness);
3. Reduce contributors to booster hesitancy.

The hypotheses, experimental procedure, and analyses were registered on OSF: https://doi.org/10.17605/OSF.IO/KUEGY (registered on 24 November 2022). The results reported here are organized into three sections according to the dependent variables: booster intention, perceptions of booster safety and effectiveness, and other contributors to booster hesitancy. All statistical tests used an alpha of 0.05 unless otherwise specified. In the interest of brevity, only those that reached significance are reported below. Prior to conducting the analyses, we examined trust in the explanations themselves to rule out differential trust. Trust in the two explanations was not significantly different (see Appendix D).

### 3.1. Booster Intention

In order to determine whether booster intention changed due to reading the explanations, a Chi-square test of independence was conducted on participants’ responses (Q8; get it ASAP, not get it, wait and see, and don’t know) by explanation condition (no-information control, effectiveness explanation, and safety explanation). Then, to better understand the impact of the explanations, as well as the impact of political ideology and pre-experimental risk perceptions and emotions on booster intentions, responses to the booster-intention question were recoded into a binary variable (“Yes” for “get it ASAP” and “No” for all other responses) and submitted to logistic regressions.

The Chi-square analysis suggested that both explanations significantly influenced booster intention (χ^2^(6) = 22.03, *p* = 0.001). As predicted, a greater proportion of participants in the explanation conditions indicated that they would get the booster as soon as possible compared to those in the no-information control condition. In addition, a smaller proportion in the explanation conditions indicated that they would wait for more information (see Table 2).

In the logistic regression conducted on the re-coded binary variable, booster intention, there were both categorical and continuous variables. The main categorical predictor was explanation condition (no-information control, effectiveness, and safety). Political ideology (liberals, moderates, and conservatives) was also included to determine whether the effect of the explanation differed depending on political ideology. To determine whether pre-experimental COVID-19 risk perception influenced booster intention, there were six continuous predictors, including likelihood ratings for contracting COVID-19 (Q1), being hospitalized (Q2), dying (Q3), and getting long COVID (Q4), as well as degree of fear (Q5) and anxiety toward COVID-19 (Q6). Note that responses to the six continuous predictors were made before the explanations were presented and were therefore unaffected by the explanations. Odds ratios for each significant predictor are reported below. Odds ratios indicate the change in the likelihood that a participant would select “get the booster ASAP” per unit change in the predictor. An odds ratio greater than one means the predictor had a positive relationship with booster intention, while an odds ratio below one indicated a negative relationship.

**Figure 1:**
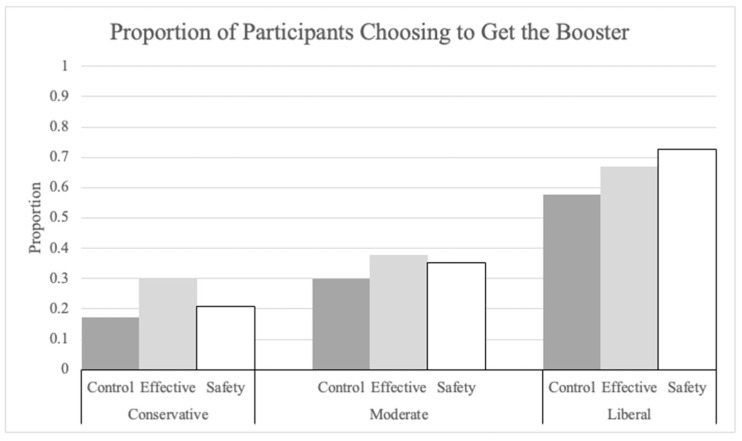
Proportion of participants choosing to get the booster by explanation condition and political ideology.

Supporting our hypothesis, and confirming the previous analysis, both explanations significantly increased participants’ intentions to get the booster. Participants with the effectiveness explanation were 1.54 (*p* = 0.003) times more likely, and those with the safety explanation were 1.77 (*p* < 0.001) times more likely, to choose to get the booster than were participants in the no-information control condition. Political ideology was also a significant predictor (Figure 1). Compared to conservatives, liberals were 5.48 times more likely (*p* < 0.001), and moderates were 1.59 times more likely (*p* = 0.028) to choose to get the booster.

Among the six continuous variables, indicating pre-experimental perceived risk of COVID-19, only fear of COVID-19 was significantly related to booster intention. For one unit increase in fear (range 0 to 100), participants were slightly more likely (1.02 times) to choose to get the booster (*p* = 0.007).

Thus, explanations of both the effectiveness and safety of the mRNA booster shot significantly increased intentions to get boosted. Political ideology was also a strong predictor of booster intention, such that liberals and moderates were more likely to choose to get the booster than conservatives. Finally, although increased fear of COVID-19 was a positive predictor of booster intentions, the effect was small, and none of the other measures of perceived risk was a significant predictor.

### 3.2. Perceptions of Effectiveness and Safety

To assess the effect of explanations on perceptions targeted in the explanations, a series of ANOVAs was conducted on the ratings of the effectiveness and safety of the booster. In all ANOVAs that were conducted and are discussed in this and the next section, the independent variables were explanation condition (no-information control, effectiveness explanation, and safety explanation) and political ideology (conservatives, moderates, and liberals). Partial Eta Squared was used to indicate the effect sizes of the omnibus tests (0.01 = small, 0.06 = medium, and 0.14 = large; [42]). Planned pairwise comparisons were made between the explanation conditions and the no-information control and between liberals and conservatives. The Holm–Bonferroni Method [43] was used in planned pairwise comparisons to correct for familywise errors. The Tukey method was used in post hoc pairwise comparisons.

#### 3.2.1. Perceived Effectiveness of the Booster

ANOVAs were conducted on effectiveness ratings for preventing contracting COVID-19 (Q10), becoming severely ill or dying from COVID-19 (Q11), and contracting new variants of COVID-19 (Q12) after being boosted. As predicted, explanations were successful in increasing perceived effectiveness of the booster in all three.

**Figure 2:**
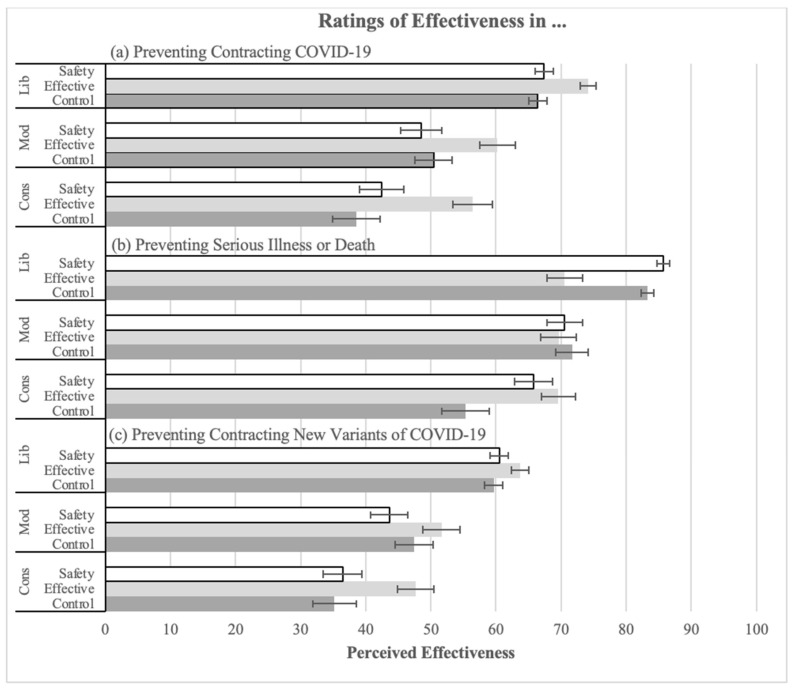
Ratings of the effectiveness of the booster in (**a**) preventing the contraction of new variants of COVID-19, (**b**) preventing serious illness or death, and (**c**) preventing contracting COVID-19 by explanation condition and political ideology.

The ANOVA on ratings of the effectiveness in preventing contracting COVID-19 (Q10) revealed a significant main effect of explanation (see Figure 2c; F(2, 1400) = 22.05, *p* < 0.001, pes = 0.03). As expected, the effectiveness explanation (M = 68.71, SD = 24.43) significantly increased ratings compared to the no-information control (M = 58.91, SD = 27.72; t(1400) = 5.97, *p* < 0.001, corrected alpha = 0.025). However, the comparison between the safety explanation and the no-information control failed to reach significance. The main effect of political ideology was significant (F(2, 1400) = 103.21, *p* < 0.001, pes = 0.13). Liberals (M = 69.37, SD = 23.24) had significantly higher mean ratings than conservatives (M = 46.16, SD = 30.06; t(1400) = 12.64, *p* < 0.001).

There was also a significant main effect of explanation on ratings for the effectiveness of the booster in preventing severe illness or death (Q11; see Figure 2b; F(2, 1400) = 5.19, *p* = 0.006, pes = 0.01). Both the effectiveness (M = 80.01, SD = 20.14; t(1400) = 3.03, *p* = 0.003 corrected alpha = 0.025) and the safety explanations (M = 79.52, SD = 22.05; t(1400) = 2.48, *p* = 0.013, corrected alpha = 0.05) significantly increased ratings compared to the no-information control (M = 76.65, SD = 23.46). The main effect of political ideology was again significant (F(2, 1400) = 127.24, *p* < 0.001, pes = 0.15). Liberals (M = 84.81, SD = 16.30) made significantly higher mean ratings than conservatives (M = 63.90, SD = 27.27; t(1400) = 14.19, *p* < 0.001). There was an interaction between the explanation and political ideology such that the explanation increased ratings mainly among conservatives (see Figure 2b; F(4, 1400) = 3.86, *p* = 0.004, pes = 0.01).

In the ANOVA on ratings of booster effectiveness in preventing contracting new variants of COVID-19 (Q12), the main effect of explanation was again significant (see Figure 2a; F(2, 1400) = 9.06, *p* < 0.001, pes = 0.01). Only the effectiveness explanation (M = 58.89, SD = 24.77) significantly increased ratings compared to the control (M = 53.46, SD = 27.15; t(1400) = 3.53, *p* < 0.001, corrected alpha = 0.025). There was also a significant main effect of political ideology (F(2, 1400) = 83.87, *p* < 0.001, pes = 0.11). Liberals (M = 61.33, SD = 23.70) had significantly higher mean ratings than conservatives (M = 40.02, SD = 26.77; t(1400) = 11.68, *p* < 0.001).

#### 3.2.2. Perceived Safety of the Booster

Next, three ANOVAs were conducted on ratings for the safety of the booster (Q9), concern about short-term side effects of the booster (Q19), and concern about long-term side effects (Q20). As predicted, explanations were successful in increasing perceived safety and reducing concern for short-term side effects.

**Figure 3:**
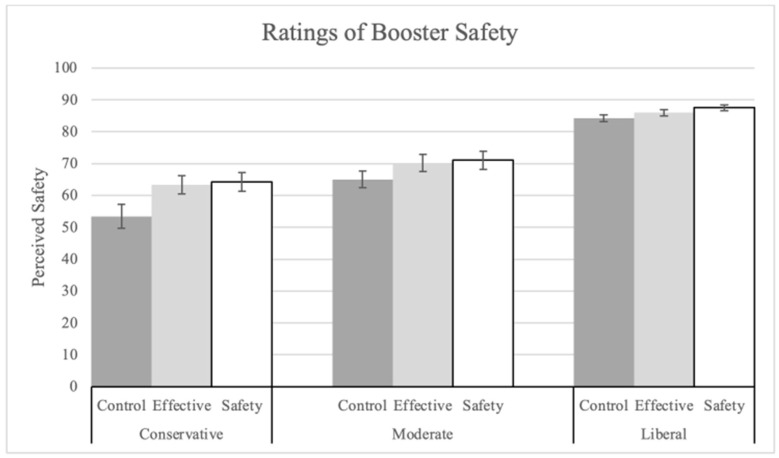
Ratings of booster safety by explanation condition and political ideology.

In the ANOVA on booster safety ratings (Q9), there was a significant main effect of explanation (see Figure 3) F(2, 1400) = 9.26, *p* < 0.001, pes = 0.01. Both the safety (M = 80.53, SD = 22.92; t(1400) = 4.02, *p* < 0.001, corrected alpha = 0.025) and the effectiveness explanations (M = 79.39, SD = 22.61; t(1400) = 3.35, *p* < 0.001, corrected alpha = 0.05) significantly increased mean ratings compared to the control (M = 75.62, SD = 25.14). Political ideology had a significant main effect (F(2, 1400) = 167.29, *p* < 0.001, pes = 0.19). Liberals (M = 85.93, SD = 17.42) made significantly higher ratings than conservatives (M = 60.59, SD = 28.18; t(1400) = 17.26, *p* < 0.001).

**Figure 4:**
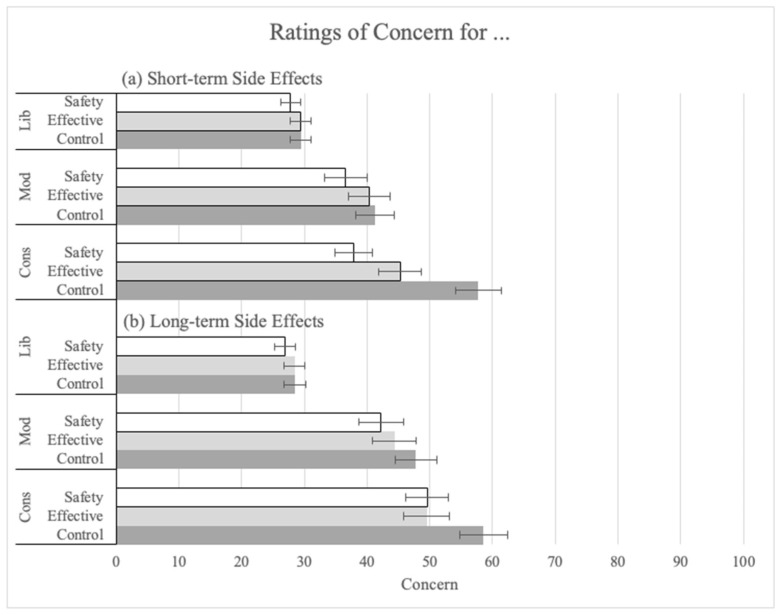
Ratings of concern for (**a**) short-term side effects and (**b**) long-term side effects by explanation condtion and political ideology.

In the ANOVA on ratings for concern about short-term side effects of the booster (Q19), there was a main effect of explanation (F(2, 1400) = 7.50, *p* < 0.001, pes = 0.01). Both the safety (M = 31.13, SD = 28.77; t(1400) = 3.87, *p* < 0.001, corrected alpha = 0.025) and the effectiveness explanations (M = 33.98, SD = 29.88; t(1400) = 1.98, *p* = 0.048, corrected alpha = 0.05) significantly reduced concern ratings compared to the no-information control (M = 36.14, SD = 30.30). Political ideology also had a significant main effect (F(2, 1400) = 42.15, *p* < 0.001, pes = 0.06). Liberals (M = 28.86, SD = 28.02) made significantly lower concern ratings than conservatives (M = 46.60, SD = 29.88; t(1400) = 8.99, *p* < 0.001). There was an interaction between the explanation and political ideology such that the explanations had a greater impact on lowering concern among conservatives than others (see Figure 4a; F(2, 1400) = 3.10, *p* = 0.015, pes = 0.01).

The main effect of explanation failed to reach significance in the ANOVA on ratings for concern about long-term side effects (Q20), not addressed in either explanation (Figure 4b). As with the previous analyses, the main effect for political ideology was significant (F(2, 1400) = 75.27, *p* < 0.001, pes = 0.10). Liberals (M = 27.92, SD = 29.72) made significantly lower concern ratings than conservatives (M = 52.32, SD = 31.38; t(1400) = 10.83, *p* < 0.001).

As predicted, the effectiveness explanation increased all three measures of effectiveness. Similarly, the safety explanation increased perceived safety and decreased perceived concern about short-term side effects. Only the decrease in concern about long-term side effects, not mentioned in the explanation, failed to reach significance.

### 3.3. Contributors to Booster Hesitancy

We next conducted ANOVAs on dependent variables operationalizing other possible contributors to booster hesitancy, including various measures of trust in the science and the scientists responsible. We also examined the potential impact on emotions (anxiety, fear, and hope) associated with the booster and its related technology. Finally, we analyzed the impact on the omission bias. All of these responses were made after the explanation manipulation and allowed us to determine whether the explanations impacted this wider range of related variables.

#### Trust in Booster Science and Scientists

ANOVAs were conducted on ratings indicating participants’ trust in the scientists who created the booster (Q14) and participants’ perception of the expertise and understanding of the scientists (Q15 and Q16). In addition, ANOVAs were conducted on ratings of participants’ perception that the booster testing was compromised (Q17) and their trust in the technology of mRNA and traditional vaccines (Q24 and 25). The explanations significantly affected all of these dependent measures.

**Figure 5:**
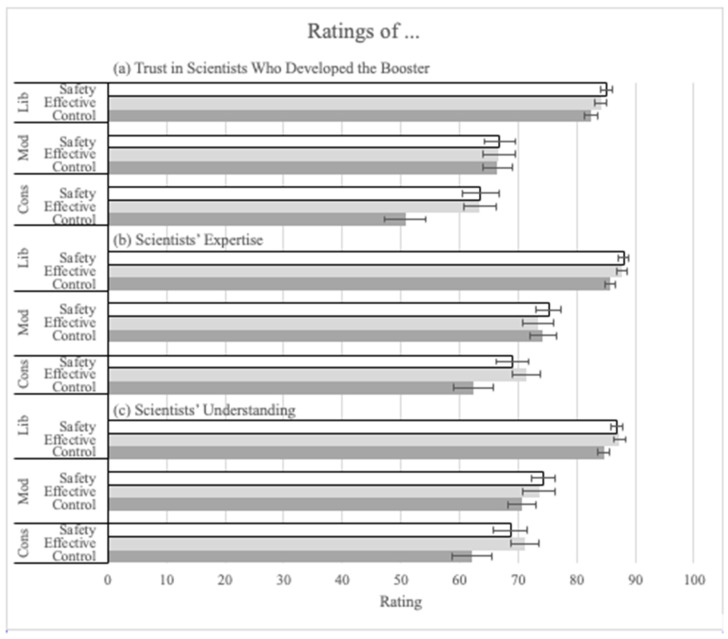
Ratings of (**a**) trust in the scientists who developed the booster, (**b**) scientists’ expertise, and (**c**) scientists’ understanding, by explanation condition and political ideology.

In the ANOVA on the trust-in-scientists ratings (Q14), there was a main effect of explanation (F(2, 1400) = 6.27, *p* = 0.002 pes = 0.01; See Figure 5a). Both the safety (M = 78.06, SD = 22.86; t(1400) = 3.19, *p* = 0.001, corrected alpha = 0.025) and the effectiveness (M = 77.60, SD = 22.60; t(1400) = 2.93, *p* = 0.004, corrected alpha = 0.05) explanations significantly increased trust compared to the control (M = 74.33, SD = 24.88). Political ideology also had a significant main effect on trust (F(2, 1400) = 159.12, *p* < 0.001, pes = 0.19). Liberals (M = 83.89, SD = 18.02) made significantly higher trust ratings than conservatives (M = 59.58, SD = 27.81; t(1400) = 15.65, *p* < 0.001). There was an interaction between explanation and political ideology such that the increase in trust ratings due to the explanations was mainly among conservatives (see Figure 5a; F(2, 1400) = 2.88, *p* = 0.021, pes = 0.01).

Next, two separate ANOVAs were conducted on participants’ ratings of the expertise and understanding of the scientists who developed the boosters. Because we were uncertain of the impact on these measures, no pre-registered hypotheses were made. Therefore, more conservative statistical procedures were used. The same is true of the measures of trust in vaccine technology. Nonetheless, the explanations increased ratings for both. There was a significant main effect of explanation on the expertise rating (Q15) (F(2, 1400) = 3.42, *p* = 0.033, pes = 0.01). Although both the effectiveness (M = 82.48, SD = 20.18) and the safety (M = 82.47, SD = 20.18) explanations increased ratings compared to the control condition (M = 79.81, SD = 21.62; see Figure 5b), none of the post hoc pairwise comparisons reached significance. As with the other analyses, the main effect of political ideology was significant (F(2, 1400) = 117.97, *p* < 0.001, pes = 0.14). The pairwise comparisons showed that liberals (M = 87.16, SD = 16.31) made significantly higher trust ratings than conservatives (M = 67.85, SD = 25.25; t(1400) = 13.73, *p* < 0.001, Tukey corrected) and moderates (M = 74.36, SD = 22.02; t(1400) = 9.65, *p* < 0.001, Tukey corrected). Moderates also made significantly higher trust ratings than conservatives (t(1400) = 3.88, *p* < 0.001, Tukey corrected).

In the ANOVA on ratings of scientists’ understanding (Q16), there was again a significant main effect of explanation (F(2, 1400) = 5.51, *p* = 0.004, pes = 0.01; see Figure 5c). Pairwise comparisons showed that both the effectiveness (M = 82.13, SD = 20.78, t(1400) = 3.07, *p* = 0.006, Tukey corrected) and the safety explanations (M = 81.46, SD = 20.54, t(1400) = 2.64, *p* = 0.023, Tukey corrected) significantly increased understanding ratings compared to the control condition (M = 78.36, SD = 22.42). The main effect of political ideology was significant (F(2, 1400) = 107.88, *p* < 0.001, pes = 0.13). Liberals (M = 86.25, SD = 17.51) made significantly higher understanding ratings than conservatives (M = 67.56, SD = 24.89; t(1400) = 12.83, *p* < 0.001, Tukey corrected) and moderates (M = 72.80, SD = 22.46; t(1400) = 9.72, *p* < 0.001). Moderates also made significantly higher ratings than conservatives (t(1400) = 3.09, *p* = 0.006, Tukey corrected).

**Figure 6:**
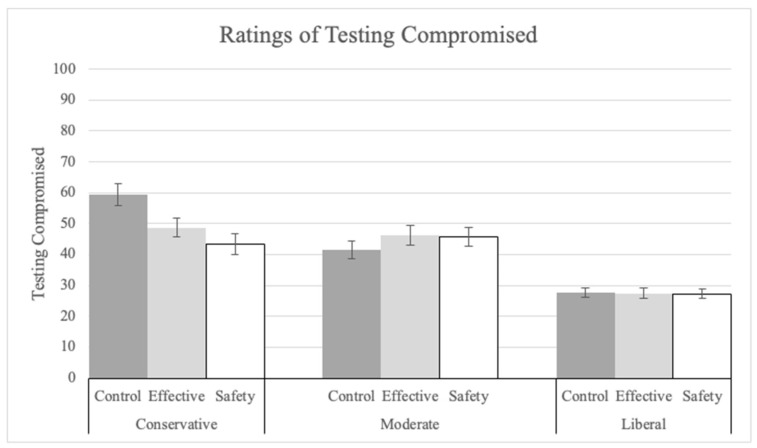
Ratings on “how much the booster testing was compromised” by explanation condition and political ideology.

Although, there was no significant main effect of explanation on participants’ ratings for agreement with the statement that the testing was compromised (Q17; see Figure 6, lower ratings indicate less compromised), there was a significant interaction between explanation and political ideology such that the ratings were most reduced among conservatives (F(4, 1400) = 3.24, *p* = 0.012, pes = 0.01). The main effect of political ideology was significant (F(2, 1400) = 81.68, *p* < 0.001, pes = 0.10). Liberals (M = 27.55, SD = 29.01) made significantly lower ratings than conservatives (M = 50.25, SD = 29.50; t(1400) = 11.00, *p* < 0.001).

**Figure 7:**
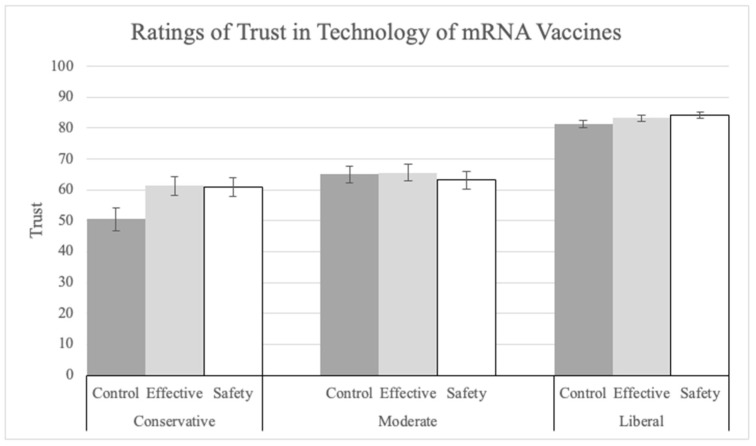
Ratings of trust in the technology of mRNA vaccines by explanation condition and political ideology.

Next, in the ANOVA on trust in the technology of mRNA vaccines (Q24), there was again a main effect of explanation (F(2, 1400) = 3.94, *p* = 0.02, pes = 0.01; see Figure 7). The effectiveness explanation (M = 76.43, SD = 23.28; t(1400) = 2.59, *p* = 0.026, Tukey corrected) increased trust ratings compared to the control (M = 73.28, SD = 25.25). Political ideology also had a significant main effect on the trust ratings (F(2, 1400) = 162.24, *p* < 0.001, pes = 0.19). Liberals (M = 82.87, SD = 18.42) made significantly higher trust ratings than moderates (M = 64.58, SD = 25.47; t(1400) = 12.08, *p* < 0.001, Tukey corrected) and conservatives (M = 57.85, SD = 28.77; t(1400) = 15.63, *p* < 0.001, Tukey corrected). Moderates also made significantly higher trust ratings than conservatives (t(1400) = 3.57, *p* = 0.001, Tukey corrected).

**Figure 8:**
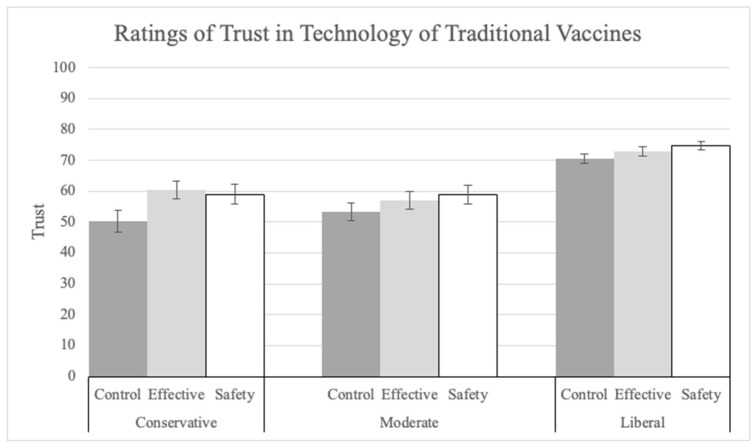
Ratings of trust in the “technology of traditional vaccines” by explanation condition and political ideology.

Somewhat surprisingly, the increase in trust conferred by the explanations, extended to traditional vaccine technology (Q25). There was a main effect of explanation (F(2, 1400) = 5.20, *p* = 0.006, pes = 0.01; see Figure 8). Both the safety (M = 69.15, SD = 27.03; t(1400) = 2.96, *p* = 0.001, Tukey corrected) and effectiveness (M = 68.08, SD = 26.75; t(1400) = 2.59, *p* = 0.026, Tukey corrected) explanations significantly increased trust compared to the control (M = 64.02, SD = 27.99). Political ideology had a significant main effect on trust ratings (F(2, 1400) = 61.77, *p* < 0.001, pes = 0.08). Liberals (M = 72.81, SD = 25.27) made significantly higher trust ratings than moderates (M = 56.37, SD = 27.64; t(1400) = 8.99, *p* < 0.001, Tukey corrected) and conservatives (M = 56.92, SD = 28.24; t(1400) = 8.30, *p* < 0.001, Tukey corrected).

### 3.4. Emotion toward the Booster

Next, ANOVAs were conducted on ratings of concern (Q18), fear (Q21), anxiety (Q22), and hope (Q23) associated with the safety and effectiveness of the booster. For these emotion ratings, unlike those mentioned above (see “Perceived Safety of the Booster” above), the safety and effectiveness addressed were generalized and referred to in the same question. Nonetheless, as predicted, the explanations reduced concern and increased hope. However, the explanations had no impact on fear or anxiety.

**Figure 9:**
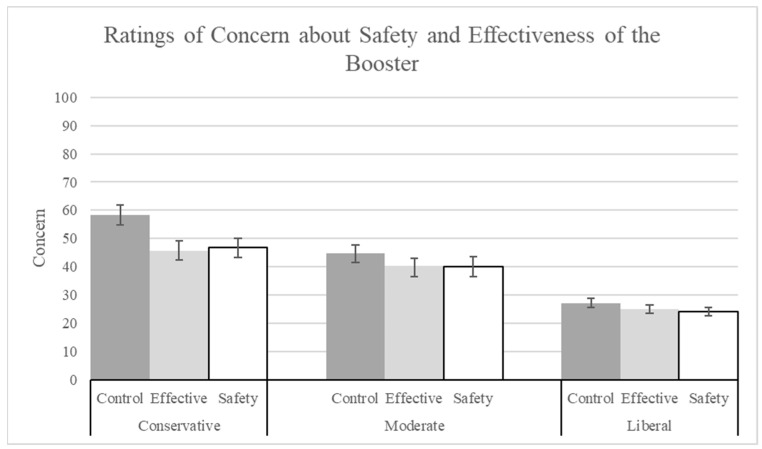
Ratings of concern for safety and effectiveness of the booster by explanation condition and political ideology.

In the ANOVA on concern (Q18), the main effect of explanation was significant (see Figure 9; F(2, 1400) = 5.56, *p* = 0.004, pes = 0.01). Both the safety (M = 30.86, SD = 29.94; t(1400) = 2.88, *p* = 0.004, corrected alpha = 0.05) and the effectiveness explanations (M = 31.12, SD = 29.34; t(1400) = 2.91, *p* = 0.004, corrected alpha = 0.025) significantly reduced concern ratings compared to the no-information control (M = 35.44, SD = 30.72). There was also a significant main effect of political ideology (F(2, 1400) = 86.25, *p* < 0.001, pes = 0.11). Liberals (M = 25.41, SD = 27.14) made significantly lower concern ratings than conservatives (M = 49.93, SD = 30.24; t(1400) = 11.79, *p* < 0.001).

**Figure 10:**
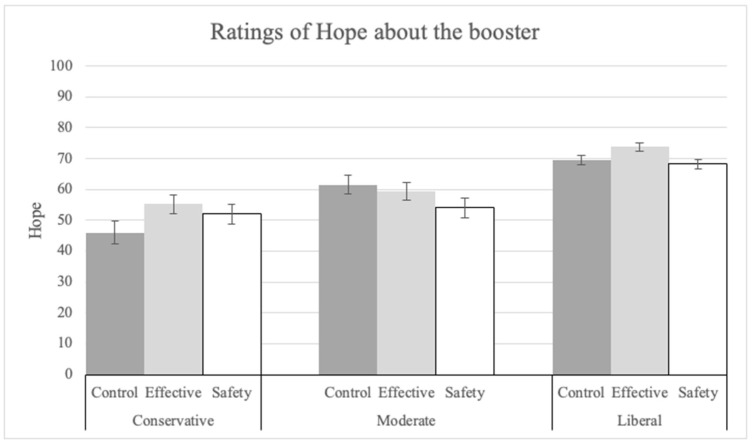
Ratings of hope about the safety and effectiveness of the booster by explanation condition and political ideology.

There was a significant main effect of explanation in the ANOVA on hope (Q23) ratings as well. Hope was rated higher in the explanation conditions compared to the control (see Figure 10; F(2, 1400) = 3.02, *p* = 0.049, pes = 0.004), although none of the pairwise comparisons reached significance. The main effect of political ideology was significant (F(2, 1400) = 61.59, *p* < 0.001, pes = 0.08). Liberals (M = 70.54, SD = 24.53) made significantly higher hope ratings than conservatives (M = 51.36, SD = 28.96; t(1400) = 10.05, *p* < 0.001).

Although the main effect of explanation failed to reach significance in the ANOVAs on fear (Q21) and anxiety (Q22) with respect to the booster, they were already fairly low. However, there was a significant main effect for political ideology on both fear (F(2, 1400) = 50.38, *p* < 0.001, pes = 0.07) and anxiety (F(2, 1400) = 35.37, *p* < 0.001, pes = 0.05). Liberals (M = 18.78, SD = 23.88) made significantly lower fear ratings than conservatives (M = 35.91, SD = 29.26; t(1400) = 9.10, *p* < 0.001). Similarly, liberals (M = 21.40, SD = 25.76) made significantly lower anxiety ratings than conservatives (M = 36.19, SD = 29.23; t(1400) = 7.49, *p* < 0.001).

The evidence presented in this section suggests that the explanations had a clear positive impact on potential contributors to booster hesitancy. Most importantly, the explanations increased trust in the scientific process, reducing the perception that the testing had been compromised, especially among conservatives; increasing trust in the technology of mRNA vaccines; and, somewhat surprisingly, increasing trust in traditional vaccines as well. Moreover, the explanations increased trust in the scientists themselves, as well as participants’ perceptions of scientists’ expertise and understanding. The positive effects of the explanations also extended to some of the emotions tested here, reducing overall concern about the boosters and increasing hope.

As predicted, political ideology played an important role such that liberals rated these issues differently than did conservatives. Nonetheless, interactions between political ideology and explanation, significant in about a quarter of the analyses, revealed that the impact of the explanations was greater among conservatives than liberals and moderates.

### 3.5. Omission Bias Question

Next, a pair of Chi-squared tests was conducted to examine the role of the omission bias (Q26). The first, on participants’ booster intention showed that the pattern of regret was different for participants who chose to get the booster ASAP compared to others. Those who chose to get the booster ASAP were much more likely to indicate that they would feel greater regret if they failed to get the booster and suffered from COVID-19 than otherwise. For the rest, the proportion in this category was smaller (χ^2^(3) = 247.96, *p* < 0.001; see Table 3).

The second Chi-square tested the potential effect of the explanations. However, participants’ pattern of regret choices was not related to explanation condition (χ^2^(6) = 5.11, *p* = 0.53; see Table 4).

Thus, it appears that those who intended to get the booster were much more likely to feel regret if they failed to get the booster and suffered from COVID-19 compared to others. However, regret was not related to explanation condition, suggesting that the explanations had no impact on the bias.

## 4. Discussion

Although almost two-thirds of Americans have received the initial doses of the COVID-19 vaccine, only a small fraction of those are up to date on the booster. This is a puzzling situation which likely has multiple causes [44]. Here, in a diverse sample of those who had received the full series of initial doses but who were not up to date on the booster despite widespread availability, we explored the impact of scientifically accurate messaging targeting two prominent reasons identified in the literature, booster side effects and vaccine effectiveness.

Indeed, both explanations significantly increased willingness to get the booster by more than 1.5 times in the effectiveness condition and almost twice as much in the safety condition. Although the effect sizes were small, it is important to note that this was a single exposure in an experimental setting. If information of this caliber were more widely available and targeted to a broader range of concerns, we might expect to see much greater increases. And yet, based on this single exposure, both explanations, including relevant statistics, were well understood by participants and significantly improved their perceptions of the both the safely and the effectiveness of the mRNA booster and vaccine. The safety explanation increased perceived safety and reduced concern about side effects. The effectiveness explanation significantly increased participants’ perception of the effectiveness of both the vaccine and the booster. Interestingly, the effectiveness explanation also increased perceived safety and decreased concern for short-terms side effects, neither of which were directly addressed in the explanation. Perhaps the bar for safety is lowered if the intervention is perceived as particularly effective, as has been seen in other situations in which context [45] or desirability [46] influence similar judgements.

However, because understanding alone does not generally account for such decisions, we also tested the impact of political ideology and pre-experimental COVID-19 risk perception. Political ideology was indeed a factor, with liberals being more than five times more willing to get the booster than constatives. On the other hand, as with some previous research [41], there was very little influence of perceived risk of COVID-19 on willingness to get the booster. Of the six pre-experimental variables tested, only fear of COVID-19 had a small but significant effect. In other words, the risk posed by the disease was not a major contributor to booster hesitancy, suggesting that other factors are critical.

Indeed, subsequent analyses demonstrated that scientifically accurate explanations addressing known concerns about booster safety and effectiveness made a difference, not only to willingness to get boosted but also to other potential contributors to booster hesitancy. Perhaps one of the most important results reported here was that the explanations increased trust, especially among conservatives, in the scientists who created the vaccine, as well as the mRNA vaccine technology. This effect extended to an increase in participants’ perception of scientists’ expertise and understanding of the issues and, among conservatives, to a reduction in the perception that the vaccine testing had been compromised [47].

Taken together, these results have implications for science communication in general. Indeed, trust has been identified as one of the most important factors in accepting and following advice in such situations [47]. In fact, the COVID-19 pandemic may have been a defining moment in the relationship between the American public and science. Recent survey results [48] suggest that trust in science took a precipitous dip during the early years of the pandemic. This underscores the challenges of science communication, especially during the science development process. Indeed, it was clear from the analyses presented here that not everyone fully appreciated the safety and effectiveness of the booster shots pre-experimentally. This was especially true among conservatives (see also [49]). Importantly, these results demonstrated that well-designed explanations can make a significant difference to perceptions of the safety and effectiveness that extend to broad increases in trust and positive effects on emotions, reducing concern and increasing hope with regard to the booster, as well as to increases in willingness to get the booster.

However, the pattern of regret revealed in responses to the omission bias [37] question was unaffected by the explanations. Nonetheless, regret was significantly different for participants who were willing to get the booster ASAP compared to others. Those willing to get the booster were much more likely to indicate that they would feel greater regret if they failed to get the booster and suffered from COVID-19 than otherwise. However, unlike some previous research in different domains [37], there was no direct evidence that those who were not boosted were affected by omission bias (preferring an error of omission). Perhaps a fruitful line of future research would be to develop and test explanations that address this issue directly.

Also absent in these results was a boomerang effect [35]. Because of the large effect of political ideology on willingness to get the booster in this sample, one might anticipate that the positive impact of the explanations would be overpowered or even reversed by the effect of political ideology. In other words, the explanations could have been ineffective or even detrimental among conservatives [36]. However, this was not the case; in fact, the oppositive was observed. Interactions between political ideology and explanation, significant in about a quarter of the ANOVAs, revealed that the improvement due to the explanations was greater among conservatives than liberals and moderates. This is not to say that explanations raised conservatives to the level of liberals. The effect of political ideology showing more booster-positive attitudes among liberals remained significant in almost all of the analyses reported here. Nonetheless, the explanations improved conservatives’ willingness to get boosted, perceptions of the safety and effectiveness of the boosters, and conservatives’ trust in the science that produced them.

Although the results of this research are compelling, there are limitations. The sample was not completely representative of the US population as a whole (e.g., slightly more highly educated, liberal, and Asian American). In addition, the primary dependent variable, willingness to get vaccinated, was based on participants’ self-reported intentions rather than their actual uptake. Previous research suggests that people do not always do what they intend to do, known as the intention–behavior gap [50,51]. There is indeed a correlation between self-reported intention and behavior; however, there are often fewer who follow through on their intentions than those who state them. In addition, the results may have been impacted by response bias. However, the strong anticipated effects of political ideology and the extension of the explanation effects to dependent variables, such as willingness to get the booster and trust in the science, suggests a genuine effect on participants’ attitudes toward the booster. Therefore, future research might seek to discover the impact of similar explanations on a more representative sample and on overt behaviors.

## 5. Conclusions

The research reported here adds to the growing evidence that understanding and trust in science go hand in hand and are a key issue in science communication. The explanations tested here were not merely persuasive, nor did they rely on scare tactics exaggerating the consequences of failing to comply. Instead, they provided complete information, expressed in everyday language, including relevant statistics that addressed participants’ concerns. In other words, the explanations tested here did not “talk down” to the reader. As such, they may have appeared honest and, as a result, trustworthy. Indeed, it is notable that not only did participants trust the information in the explanations provided, as well the testing process and the mRNA technology itself, but that trust extended to the scientists who developed the vaccines, as well as to perceptions of scientists’ expertise and understanding of the issues.

As such, the lessons learned here are relevant for the current circumstance but also for future situations in which people need to trust recommendations based on science, including emerging science. This research and others like it [11,23,24] suggest that risk communication, which provides understandable but complete scientific explanations targeting known concerns, is capable of maintaining and even increasing trust, as well as providing critical information upon which to make informed decisions in the face of future risks.

## Figures and Tables

**Table 1:** Explanations provided in the experimental conditions.

**Condition**	**Explanation**
No-Information Control	
Condition 1: Effectiveness	The following explanation refers to mRNA vaccines such as Pfizer and Moderna: The vaccine is 86% effective in preventing symptomatic infection by COVID-19 for the first 5 months. Without the booster shot, effectiveness drops **to 42% in month 6.** Booster shots at 5 months are recommended by the CDC/by the pharmaceutical company that produces the vaccine. With a booster shot, the vaccine is **90% effective.**
Condition 2: Safety	The following explanation refers to mRNA vaccines such as Pfizer and Moderna: Of the side effects reported for over 12,591 booster shots of the vaccine, **99% were minor**. They were comparable to those experienced with the second of the initial doses. Symptoms: muscle pain and swelling at the injection site, reported by about 80%. fever and headache reported by 74%. All of these common side effects are signs that the vaccine is working and stimulating your immune system.

**Table 2:** Contingency table with booster intention and the explanations.

Booster Intention	Explanations		
	No-Info Control	Effectiveness	Safety
I would get an (or another if I already have one) mRNA COVID-19 booster shot as soon as possible	214 (46%)	262 (56%)	269 (57%)
I would wait to get an (or another if I already have one) mRNA COVID-19 booster shot until there is more information	130 (28%)	92 (20%)	78 (17%)
I don’t know	36 (8%)	35 (7%)	36 (8%)
I would not get an (or another if I already have one) mRNA COVID-19 booster shot	86 (18%)	82 (17%)	89 (19%)

**Table 3:** Frequency of responses to the regret question by booster intention.

Regret Option	Booster Yes	Booster No
More regret if boosted	24 (3%)	127 (19%)
More regret if un-boosted	539 (72%)	219 (33%)
Same regret	100 (13%)	220 (33%)
No regret	82 (11%)	98 (15%)

**Table 4:** Frequency of responses to the regret question by explanation condition.

Regret Option	Control	Effectiveness	Safety
More regret if boosted	55 (12%)	49 (10%)	47 (10%)
More regret if un-boosted	245 (53%)	251 (53%)	262 (56%)
Same regret	103 (22%)	119 (25%)	98 (21%)
No regret	63 (14%)	52 (11%)	65 (14%)

**Figure A1:**
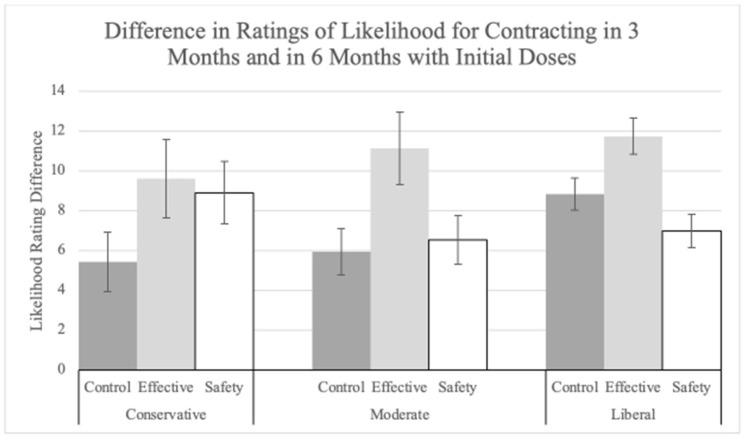
Difference in ratings of likelihood of contracting COVID-19 in the first three months with initial doses and contracting in the first six months by explanation condition and political ideology.

**Figure A2:**
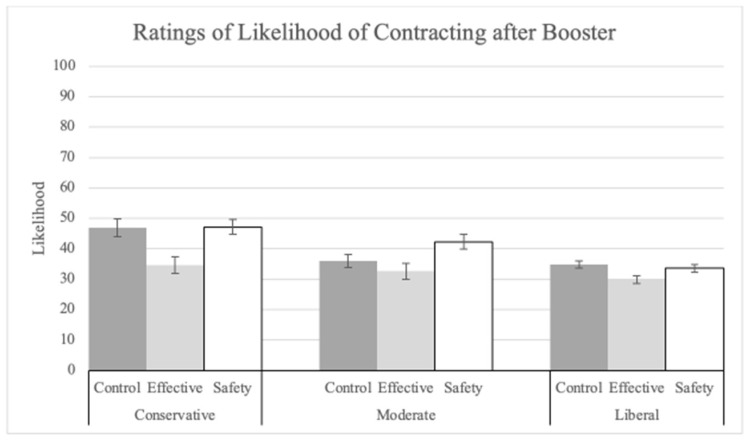
Ratings of likelihood of contracting COVID-19 with the booster by explanation condition and political ideology.

**Figure A3:**
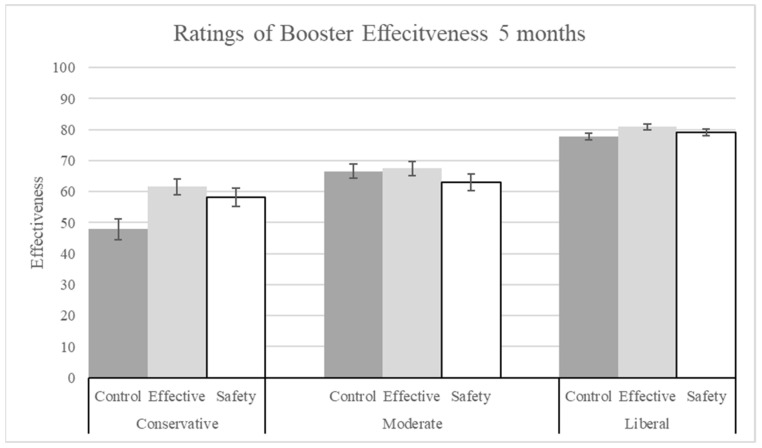
Ratings of effectiveness of the booster in the first five months by explanation condition and political ideology.

## Data Availability

Data, analytic codes, and materials for the experiments reported here are available on Open Science Framework Pre-Registration or upon request. The link to the pre-registration is https://doi.org/10.17605/OSF.IO/KUEGY (registered on 24 November 2022).

## Appendix A. Demographic Data

**Table A1:** Age compared to the general population.

	US 2020	This Experiment	
Age Group	Percentage	N	Percentage
18–49	77.9%	1162	82%
50+	~31%	247	18%

Note. US 2020 source: US Census Bureau [52,53].

**Table A2:** Education compared to the general population.

	US 2021	This Experiment
Education	Percentage	Percentage
Lower than bachelor’s	67.10%	43%
Bachelor’s or higher	32.90%	56%

Note. US 2021 source: US Census Bureau [54].

**Table A3:** Race and ethnicity.

	US 2021	This Experiment	
Race and Ethnicity	Percentage	N	Percentage
African American, Black, African, Caribbean	13.40%	115	8%
Asian American, Asian, Pacific Islander	5.90%	139	10%
Bi-racial, multi-racial	2.8%	51	4%
European American, White, Anglo, Caucasian	60.1%	979	69%
Hispanic American, Latina(o), Chicana(o)	18.50%	104	7%
Middle Eastern/North African	NA	9	1%
Native American, American Indian	1.30%	3	0%
Other	NA	1	0%
Prefer not to answer	NA	8	1%

Note. US 2021 source: US Census Bureau [54].

**Table A4:** Political ideology.

	US 2021	This Experiment	
Political Ideology	Percentage	N	Percentage
Conservative	37%	227	16%
Moderate	36%	270	19%
Liberal	25%	912	65%

Note. US 2021 source: Saad [55].

**Table A5:** Results of logistic regression for willingness to get booster shot with sociodemographic variables and explanation conditions.

Variables	Levels	Estimate	Odds Ratio	*p*-Value	N
Continuous					
Age		0.009	1.09	0.09	
Categorical					
Gender	Female	Reference			642
	Male	0.03	1.03	0.82	738
	Other	0.52	1.68	0.25	29
Education	Bachelor’s degree	Reference			584
	No high school	14.97	3,187,205	0.96	6
	High school diploma	−0.22	0.08	0.26	179
	Associate’s degree	0.08	1.08	0.57	427
	Master’s degree	0.24	1.27	0.21	171
	Doctorate degree	−0.05	0.95	0.88	38
	Prefer not to answer	−0.86	0.42	0.49	4
Ethnicity	White American	Reference			979
	African American	−0.16	0.85	0.47	115
	Asian American	−0.12	0.89	0.57	139
	Bi-racial	−0.09	0.92	0.78	51
	Hispanic American	0.03	1.03	0.91	104
	Middle Eastern	0.06	1.06	0.93	9
	Native American	1.01	2.75	0.49	3
	Other	−14.93	<0.001	0.99	1
	Prefer not to answer	−1.13	0.32	0.23	8
Annual income	Less than USD 10,000	Reference			65
	USD 10,000–USD 19,999	0.12	1.13	0.74	81
	USD 20,000–USD 29,999	−0.08	0.92	0.81	127
	USD 30,000–USD 39,999	0.31	1.37	0.34	133
	USD 40,000–USD 49,999	0.25	1.28	0.46	128
	USD 50,000–USD 59,999	−0.09	0.91	0.78	141
	USD 60,000–USD 69,999	−0.13	0.88	0.71	110
	USD 70,000–USD 79,999	−0.29	0.75	0.41	100
	USD 80,000–USD 89,999	0.26	1.30	0.47	93
	USD 90,000–USD 99,999	0.04	1.05	0.90	90
	USD 100,000–USD 149,999	0.09	1.10	0.77	191
	USD 150,000 or more	0.16	1.18	0.64	124
	Prefer not to answer	0.23	1.26	0.67	26
Political ideology	Conservative	Reference			466
	Liberal	2.02	7.55	<0.001	471
	Moderate	0.7	2.02	<0.001	472
Condition	Control	Reference			227
	Effectiveness	0.45	1.57	0.002	912
	Safety	0.54	1.72	<0.001	270

## Appendix B. Experiment Questions

**Question Number**	**Question Wording**
1	How likely do you think it is that you would become **infected** with COVID-19 in the next 6 months?
2	If you become infected with COVID-19, how likely do you think it is that you would **die** from COVID-19
3	If you become infected with COVID-19, how likely do you think it is that you would be **hospitalized**?
4	If you become infected with COVID-19, how likely do you think it is that you would experience “**long COVID-19**” symptoms?
5	When thinking about becoming infected with COVID-19, how much **fear** do you feel?
6	When thinking about becoming infected with COVID-19, how much **anxiety** do you feel?
7	Choose the statement that best describes **your status** with regard to the mRNA COVID-19 booster shots. (1) I am not fully vaccinated with the initial doses of the vaccine, and, therefore, I am not eligible for booster shots. (2) I am fully vaccinated with the initial doses, but I have received none of the mRNA COVID-19 booster shots for which I am eligible. (3) I am fully vaccinated with the initial doses, and I have already received some but not all mRNA COVID-19 booster shots for which I am eligible. How many have you received [number]? (4) I am fully vaccinated with the initial doses, and I have already received all the mRNA COVID-19 booster shots for which I am eligible. How many have you received [number]?
8	If you have not received all the mRNA COVID-19 booster shots for which you are eligible, choose the statement that best describes your situation or intentions. (1) I would get an (or another if I already have one) mRNA COVID-19 booster shot as soon as possible. (2) I would wait to get an (or another if I already have one) mRNA COVID-19 booster shot until there is more information. (3) I would not get an (or another if I already have one) mRNA COVID-19 booster shot. (4) I don’t know.
9	How **safe** do you think the mRNA COVID-19 booster shots are? *[VAS endpoints “Not at all safe” to” Completely safe”]*
10	How effective do you think the mRNA COVID-19 booster shots are in preventing you from **contracting COVID-19** if you were exposed to it? *[VAS endpoints “Not at all effective” to” Completely effective”]*
11	How effective do you think the mRNA COVID-19 booster shots are in preventing **severe illness or death** if you were exposed to COVID-19 and contracted it? *[VAS endpoints “Not at all effective” to” Completely effective”]*
12	How effective do you think the mRNA COVID-19 booster shots are in preventing you from **contracting COVID-19** if you were exposed to one of the **new variants** of COVID-19? *[VAS endpoints “Not at all effective” to” Completely effective”]*
13	How much do you **trust the information** about COVID-19 that is provided above? *NOTE: This question is NOT shown to those participants in the no-information control group.* *[VAS endpoints “Not at all” to” Completely”]*
14	How much do you **trust the scientists** who developed and tested mRNA COVID-19 vaccine boosters? *[VAS endpoints “Not at all” to” Completely Trust”]*
15	Thinking about the scientists who developed and tested the mRNA COVID-19 vaccine boosters: How much of the necessary **expertise** do they have to develop a safe and effective COVID-19 vaccine booster? *[VAS endpoints “None” to “All”]*
16	Thinking about the scientists who created and tested the mRNA COVID-19 vaccine boosters: How well do they **understand the issues** relevant to developing a safe and effective COVID-19 vaccine booster? *[VAS endpoints “Not at all” to” Completely”]*
17	Thinking about the scientists who created and tested the mRNA COVID-19 vaccines: To what extent do you think they **compromised the testing** of the COVID-19 vaccine boosters (cut corners) to make it quickly available? *[VAS endpoints “Not at all” to” Completely”]*
18	How concerned are you about the **safety and effectiveness** of mRNA COVID-19 booster shots? *[VAS endpoints “Not at all concerned” to” Extremely concerned”]*
19	How concerned are you about any possible **short-term side effects** of mRNA COVID-19 booster shots? *[VAS endpoints “Not at all concerned” to” Extremely concerned”]*
20	How concerned are you about any possible **long-term side effects** of mRNA COVID-19 booster shots? *[VAS endpoints “Not at all concerned” to” Extremely concerned”]*
21	When thinking about the safety and effectiveness of mRNA COVID-19 booster shots, how much **fear** do you feel? *[VAS endpoints “None” to” A Lot”]*
22	When thinking about the safety and effectiveness of mRNA COVID-19 booster shots, how much **anxiety** do you feel? *[VAS endpoints “None” to” A Lot”]*
23	When thinking about the safety and effectiveness of mRNA COVID-19 booster, how much **hope** do you feel? *[VAS endpoints “None” to” A Lot”]*
24	How much do you **trust the technology of mRNA vaccines** like Pfizer and Moderna? *[VAS endpoints “Not at all” to “Completely”]*
25	How much do you **trust the technology of conventional vaccines** like Johnson & Johnson? *[VAS endpoints “Not at all” to “Completely”]*
26	In which situation do you think you will feel more **regret**? (1) You chose to get the booster and you suffered severe side effects of the booster shot. (2) You chose not to get the booster shot and you suffered from severe illness from COVID-19. (3) Same amount of regret in both cases. (4) No regret.
27	How likely do you think it is that you would become infected with COVID-19 in the **first 3 months** after being fully vaccinated with the initial doses of the mRNA COVID-19 vaccine (but not boosted)? *[VAS endpoints “Impossible” to “Certain”]*
28	How likely do you think it is that you would become infected with COVID-19 **6 months after** being fully vaccinated with the initial doses of the mRNA COVID-19 vaccine (but not boosted)? *[VAS endpoints “Impossible” to “Certain”]*
29	What do you think is the **effectiveness of the mRNA vaccines for the first 5 months** after vaccination? *[VAS endpoints “Not effective all” to “Extremely Effective”]*
30	How likely do you think it is that you would become infected with COVID-19 after being fully vaccinated with the initial doses of the mRNA COVID-19 vaccine and **boosted**? *[VAS endpoints “Impossible” to “Certain”]*
31	Gender (Options: Male, Female, Other)
32	Age (in years): numeric
33	What is the highest degree or level of school that you have completed? Please select ONE option. (1) Did not complete high school (2) High school diploma or GED equivalent (3) Some college, technical school, or associate’s degree (4) Bachelor’s degree (e.g., BA, BS) (5) Master’s degree (e.g., MA, MS, MEng, MEd, MSW, MBA) (6) Professional degree or doctorate (e.g., MD, DDS, DVM, LLB, JD, PhD, EdD)
34	Please select ALL that apply to you. (1) African American, Black, African, Caribbean (2) Asian American, Asian, Pacific Islander (3) European American, White, Anglo, Caucasian (4) Hispanic American, Latina(o), Chicana(o) (5) Native American, American Indian (6) Middle Eastern/North African (7) Bi-racial, Multi-racial (8) Other (9) Prefer not to answer.
35	Please indicate the answer that includes your entire household income in (previous year) before taxes. Select one from: (1) less than $10,000 (2) $10,000–$19,999 (3) $20,000–$29,999 (4) $30,000–$39,999 (5) $40,000–$49,999 (6) $50,000–$59,999 (7) $60,000–$69,999 (8) $70,000–$79,999 (9) $80,000–$89,999 (10) $90,000–$99,999 (11) $100,000–$149,999 (12) $150,000 or more (13) Prefer not to answer.
36	Political ideology: In general, do you think of yourself as: (1) Extremely Liberal (2) Liberal; Slightly Liberal (3) Moderate, middle of the road (4) Slightly conservative (5) Conservative (6) Extremely Conservative
37	We just want to make sure you are paying attention. This study is about COVID-19, but we want to you choose Ebola for this question. (1) COVID-19. (2) Cancer. (3) Ebola. (4) Diabetes. *Note: Participants not answering Ebola are removed from further analyses.*

Please note that there were additional questions relevant to a separate project included in the survey, but the responses to these questions are not reported here.

## Appendix C. Understanding Effectiveness Explanation

In order to determine whether participants understood the duration of the vaccine protection and the renewed protection provided by the booster addressed in the efficacy explanation, seven unregistered ANOVAs, using an alpha level of 0.05, and accompanying Tukey-corrected post hoc pairwise comparisons were conducted on participants’ ratings of the likelihood of getting infected with COVID19 at 3 and 6 months post-initial vaccination (Q27 and Q28), getting infected after being boosted (Q29), and the effectiveness of the vaccines within the first five months (Q30). The independent variables in these analyses were explanation condition (no-information control, effectiveness, and safety) and political ideology (conservatives, moderates, and liberals).

First, the likelihood rating for infection at six months (Q28) was subtracted from the rating for three months (Q27). Then, an ANOVA on the difference score was conducted, with a greater difference indicating greater effectiveness at three months. Indeed, there was a significant main effect of explanation (see Figure A1; F(2, 1400) = 7.31, *p* < 0.001, pes = 0.01). Those with the effectiveness explanation had a significantly greater mean difference score (M = 11.28, SD = 16.23) than those with both the safety explanation (M = 7.21, SD = 13.92; t(1400) = 2.96, *p* = 0.008, Tukey corrected) and those in the control condition (M = 7.72, SD = 13.22; t(1400) = 3.58, *p* = 0.001, Tukey corrected). Neither the main effect of political ideology nor the interaction reached significance.

Next, in the ANOVA on the ratings of likelihood of contracting COVID-19 after receiving the booster, there was again a significant main effect of explanation (see Figure A2; F(2, 1400) = 7.93, *p* < 0.001, pes = 0.01). Those with the effectiveness explanation (M = 31.17, SD = 22.70) made significantly lower ratings than those in both the safety explanation (M = 37.50, SD = 23.19; t(1400) = 4.93, *p* < 0.001, Tukey corrected) and the control conditions (M = 36.90, SD = 22.24; t(1400) = 3.89, *p* < 0.001, Tukey corrected). There was a significant main effect of political ideology (F(2, 1400) = 19.66, *p* < 0.001, pes = 0.03). Liberals (M = 32.75, SD = 22.15; t(1400) = 6.10, *p* < 0.001, Tukey corrected) and moderates (M = 37.11, SD = 23.02) made lower ratings than conservatives (M = 42.69, SD = 23.82); t(1400) = 2.94, *p* = 0.009, Tukey corrected). Liberals also made lower ratings than moderates (t(1400) = 2.71, *p* = 0.019, Tukey corrected).

Next, in the ANOVA on ratings of the effectiveness of the booster in the first five months, there was, again, a significant main effect of explanation (see Figure A3; F(2, 1400) = 6.92, *p* < 0.001, pes = 0.01). Those with the effectiveness explanation (M = 75.30, SD = 20.10) made significantly higher ratings than did those in the control condition (M = 71.02, SD = 23.78; t(1400) = 3.71, *p* = 0.001, Tukey corrected). There was a significant main effect of political ideology (F(2, 1400) = 143.48, *p* < 0.001, pes = 0.17). Liberals (M = 79.32, SD = 17.54) made significantly higher ratings than moderates (M = 65.66, SD = 23.35; t(1400) = 9.71, *p* < 0.001, Tukey corrected) and conservatives (M = 65.66, SD = 23.35; t(1400) = 15.63, *p* < 0.001, Tukey corrected). Moderates also made higher ratings than conservatives (t(1400) = 5.39, *p* < 0.001, Tukey corrected). There was also a significant interaction between explanation and political ideology, F(2, 1400) = 3.45, *p* = 0.008, pes = 0.01. The effect of explanation was mainly among conservatives. Those with both the safety (M = 58.21, SD = 25.93; t(1400) = 3.11, *p* = 0.006, Tukey corrected) and the effectiveness explanations (M = 61.61, SD = 22.81; t(1400) = 4.17, *p* < 0.001, Tukey corrected) made higher ratings than those in the control condition (M = 47.86, SD = 27.73).

## Appendix D. Analyses on Trust in Explanations

An ANOVA conducted on trust in the explanation ratings, including only the effectiveness and safety explanation conditions, revealed that mean trust in the safety explanation (M = 77.72, SD = 23.37) was not significantly different from that of the effectiveness explanation (M = 75.13, SD = 23.80; F(2, 937) = 89.14, *p* < 0.001, pes = 0.002). There was a significant main effect of political ideology on trust (F(2, 937) = 89.14, *p* < 0.001, pes = 0.16). Liberals (M = 83.13, SD = 18.29) had higher mean trust ratings than conservatives (M = 59.99, SD = 28.19; t(937) = 11.94, *p* < 0.001, Tukey corrected) and moderates (M = 67.57, SD = 25.62; t(937) = 8.37, *p* < 0.001, Tukey corrected). Moderates had significantly higher mean trust ratings than conservatives (t(937) = 3.14, *p* = 0.005, Tukey corrected).
